# Some of Our Medical Universities and Colleges

**Published:** 1880-04

**Authors:** 


Some of our Medical Universities and Colleges.—The oldest and most distinguished of our universities seem to be keeping step *pari passu* with the increase of knowledge and development of the resources of the times in which we live. That knowledge is power is not a debatable question even in these degenerate days, when mammon seems to be the god of the sensualist and the skeptic, and sways a sceptre that even the well-born and carefully cultured cannot entirely ignore, whilst its potency and influence over the masses is overwhelming and complete. Without money, however, it is of course impossible to erect suitable buildings, furnish and equip chemical and other laboratories, establish museums, etc. But when these are prepared and ready for use, brains and culture must be brought into requisition to render them of value in the impartation of knowledge. It is pleasing to observe the rapid strides that have been taken in the last few years, and see the vastly increased and continuous augmentation of facilities that some of our older universities now offer for the intellectual culture and improvement of the youth of the present time. The University of Pennsylvania, the first, chronologically, in its medical department, in this country, has ever held high rank among the leading medical schools of the world; has its medical, art and literary, law, philosophical, and dental departments all thoroughly organized and equipped, and officered by men, for the most part, of distinguished ability and cosmopolitan reputation. The high standard it has maintained for admission to the degree of Doctor of Medicine has imparted an intrinsic value to its diploma that inspires confidence and respect wherever it is known. The thoroughness of its course of instruction, extending over three sessions, is not surpassed anywhere.

Harvard University is likewise an institution of great thoroughness in its several departments, which embrace schools of medicine, art, law, theology and dentistry. These are presided over by men of eminence and standing in their several departments and professorships.

The Universities of Maryland and New York City have each their medical and literary departments, and the former schools are of extended celebrity for the soundness of their teaching. Besides these universities there are several colleges that have medical schools attached to them. In addition to those there are the Jefferson College of Philadelphia, and the College of Physicians and Surgeons of New York, that were established originally as medical schools or departments of literary colleges. Both of them are, however, now, we believe, independent institutions, and, like the Bellevue Medical College of New York, are of extended and deservedly high reputation for the ability and learning of their professors and thoroughness of their courses of instruction. There are still other universities and colleges well entitled to places on this distinguished and honorable list of medical institutions, but want of space will not allow of our naming them now. In a future issue we hope to call attention to the more prominent medical institutions of the South and West.

